# Developing indicators for measuring low-value care: mapping *Choosing Wisely* recommendations to hospital data

**DOI:** 10.1186/s13104-018-3270-4

**Published:** 2018-03-05

**Authors:** Kelsey Chalmers, Tim Badgery-Parker, Sallie-Anne Pearson, Jonathan Brett, Ian A. Scott, Adam G. Elshaug

**Affiliations:** 1 Menzies Centre for Health Policy, School of Public Health, Charles Perkins Centre, University of Sydney, Sydney, NSW 2006 Australia; 2 Health Market Quality Program, Capital Markets Cooperative Research Centre, Sydney, NSW 2000 Australia; 3 Medicines Policy Research Unit, Centre for Big Data Research in Health, University of New South Wales, Level 1, AGSM Building, Sydney, NSW 2052 Australia; 4 Princess Alexandra Hospital, Brisbane, QLD 4102 Australia; 5 University of Queensland, Brisbane, QLD 4072 Australia

**Keywords:** Inappropriate care, Low-value care, Quality measurement, Choosing Wisely, Hospitals, Quality of health care, Disinvestment

## Abstract

**Objective:**

Low-value health care refers to interventions where the risk of harm or costs exceeds the likely benefit for a patient. We aimed to develop indicators of low-value care, based on selected *Choosing Wisely* (CW) recommendations, applicable to routinely collected, hospital claims data.

**Results:**

We assessed 824 recommendations from the United States, Canada, Australia and the United Kingdom CW lists regarding their capacity to be measured in administrative hospital admissions datasets. We selected recommendations if they met the following criteria: the service occurred in the hospital setting (*observable in setting*); a claim recorded the use of the service (*record of service*); the appropriate/inappropriate use of the service could be mapped to information within the hospital claim (*indication*); and the service is consistently recorded in the claims (*consistent documentation*). We identified 17 recommendations (15 services) as measurable. We then developed low-value care indicators for two hospital datasets based on the selected recommendations, previously published indicators, and clinical input.

## Introduction

Low-value care offers limited or no benefit and poses unnecessary risks and costs to patients [1, 2]. Publishing “do-not-do” recommendations, which detail inappropriate services for patient groups, is a recent strategy to redress low-value care. An example of this is the international Choosing Wisely (CW) campaign [3].

One way to assess low-value care is to develop direct measures in routinely collected data [4]. These datasets, including health insurance claims and hospital administrative databases, provide population-level insights on service utilisation. Direct measures of low-value care use information collected at the individual patient level to distinguish inappropriate from appropriate care [5]. This approach contrasts with measuring low-value care indirectly via geographic variation in services, which does not necessarily distinguish unwarranted from warranted variation. Using these direct measures is an additive approach to measuring low-value care, as described by Miller et al. [6], and is suitable for both patient- and service-centric measures of low-value care [7].

We isolated CW recommendations with characteristics conducive to indicator development, thus allowing for direct measurement in routinely collected data on inpatient admissions. While these indicators are not formal quality metrics, which would require further investigation of their reliability and validity, they can provide an estimate of low-value services used within health care systems.

## Main text

### Methods

#### Datasets

Our two datasets were (1) hospital and medical claims from a group of Australian private health insurance (PHI) funds, and (2) public hospital admissions data from Australia’s most populous state (New South Wales). Australians can receive care in public or private hospitals, and care is funded by some combination of patients’ out-of-pocket payments, their PHI, and the government [8]. Both datasets include information on inpatient admissions, with details such as the patient’s age, gender, procedures during the admission, and diagnoses coded at discharge. The datasets cover different populations (although not mutually exclusive), and are therefore not directly comparable.

The PHI dataset contains medical and hospital claims made to 13 Australian insurance funds, as well as the Hospital Casemix Protocol data, sent from hospitals to these insurance funds after a patient’s discharge and used by the Australian government and insurance industry [9]. Clinical coders record medical services using the Medicare Benefits Scheme (MBS) item numbers (if claimed by a clinician to the insurance fund) and the Australian Classification of Health Interventions (ACHI). Within the public hospital dataset, this MBS code is not present since clinicians are not paid per service use. Both datasets have diagnosis information coded using the International Classification of Diseases—10th revision—Australian Modification (ICD-10-AM).

Clinical information used in forming the diagnosis, such as results of pathology tests or imaging, is not available in either dataset. Prescribing information and pathology requests are also not included.

#### Criteria for whether a recommendation can be measured

We developed four criteria to consistently and transparently select recommendations measurable in the two datasets used in our work. The specific criteria and their applied order exclude the least relevant (to the dataset) recommendations first, leaving those recommendations requiring further investigation and resources to determine their measurability.

i. *Observable in setting* The recommendation is relevant to the dataset and its health care setting.

We excluded recommendations related to procedures or services unlikely to occur in an inpatient setting, such as those usually performed in general practice.

ii. *Record of service in claim* The service can be recorded in the claim as the relevant and specific code exists for it.

Australian inpatient claims include procedures but not the prescribing of medicines. Hence, recommendations related to in-hospital prescription drugs are not measurable in claims data.

iii. *Indication* It is possible to map the appropriate/inappropriate patient criteria for a service described in the recommendation to the information within the claim.

Sometimes the caveats on the recommended inappropriate use of a service are too non-specific compared with the clinical detail recorded in the claims. For example, we cannot investigate a recommendation of the form “do not do … without careful consideration.” A *CW Australia* recommendation states that inguinal hernia repair should not occur: “…without careful consideration, particularly in patients who have significant co-morbidities” [10]. A direct measure of low-value care based on this recommendation would arguably have poor specificity, because it would label too many appropriate inguinal hernia repairs as low-value.

iv. *Consistent documentation* The service is consistently recorded in the claim according to standard coding practices.

We cannot measure a recommendation across a population if the service is not consistently documented during the data collection, as the service count will be underreported. In some cases, a radiologist will claim for an imaging service for an admitted patient, so the relevant MBS item will be in the private claims dataset. However, the Australian Coding Standards for ACHI coding classify imaging services as ‘procedures not normally coded’, because “they are usually routine in nature, performed for most patients and/or can occur multiple times in an episode” [11]. Some low-value care described by these recommendations might be identifiable, and so these measures might be of interest to payers, but they are not generalisable because the estimates would be under-representative and possibly biased.

#### Selecting measurable recommendations in hospital admissions data

**Fig. 1:**
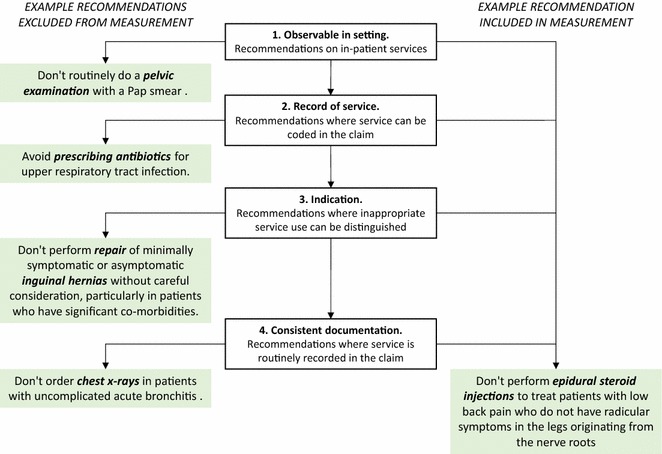
Criteria for direct measurement of Choosing Wisely recommendations, with example recommendations from Choosing Wisely Australia [10]. Recommendations are excluded in four stages, as described by the steps in the middle column. Four examples of excluded Choosing Wisely Australia recommendations are shown in the left column. Pap smear was excluded as this is a service usually provided in general practice. Antibiotic prescription was excluded as prescribing is not recorded within either the private or public available datasets. Inguinal hernia repair was excluded because the description of “minimally symptomatic or asymptomatic” or “careful consideration” is not recorded in the data. Finally, chest x-rays were excluded as this is a service that is not usually coded in inpatient data, according to the *Australian Coding Standards* [11]. The example of the epidural steroid injection recommendation meets all four criteria for measurement

We investigated 824 recommendations from CW lists in the US, Canada, Australia, and the UK, downloaded in January 2017 [12–15]. Two authors (KC, TBP) reviewed the recommendations independently to assess measurability in the private and public datasets, and then resolved discrepancies by consensus. Figure 1 illustrates this review process according to the measurement criteria.

#### Adapting recommendations for measurement

We followed an approach used by Schwartz et al. [16] and further described in Brett et al. [17], and defined a narrow indicator (more specific, which might exclude some low-value care) and a broad indicator (more sensitive, which might include some appropriate care) for each service when necessary. Where they exist, we used previously published indicators of these low-value services as a starting point [16, 18] and adapted these based on the details in our data and health care setting, or modified them in response to clinical advice or updates to recommendations.

Even when a recommendation was measureable according to the criteria there could still be ambiguity on how to define low-value service use. For example, multiple recommendations relating to the same service were sometimes slightly different, such as two CW US recommendations on inferior vena caval filter use from the Society for Vascular Surgery and the American Society of Hematology.

For some indicators, we developed a proxy measure for the “asymptomatic” patient groups. We excluded patients with diagnoses related to possible symptoms (see definitions for carotid endarterectomy and endovascular repair of abdominal aortic aneurysms in Table 1).

**Table 1:** Fourteen operational definitions adapted from 18 Choosing Wisely recommendations to hospital claims data

Recommendation (source)	Narrower definition	Broader definition
Avoid performing a colonoscopy for constipation in those under the age of 50 years without family history of colon cancer or alarm features (CWC)	Colonoscopy with diagnosis of constipation, and no diagnoses of anaemia, weight loss, family or personal history of cancer of digestive system, or personal history of other diseases of the digestive system in previous 12 months. Minimum age: 18. Maximum age: 49. Sex: both	Colonoscopy with diagnosis of constipation and no anaemia, weight loss, or family or personal history of cancer of the digestive system recorded in the admission. Minimum age: 18. Maximum age: 49. Sex: both
Don’t perform carotid endarterectomies or stenting in most asymptomatic high-risk patients with limited life expectancy (CWC)	Carotid endarterectomy with no stroke or focal neurological symptoms recorded, and ASA code 4–5 or age 75 + with ASA 3. Exclude emergency admissions and admissions from the emergency department. Minimum age: 18. Sex: both [16, 26]	Carotid endarterectomy with no stroke or focal neurological symptoms recorded, and ASA code 4–5 or age 75 +. Minimum age: 18. Sex: both [16, 26]
Don’t perform endovascular repair of abdominal aortic aneurysms in most asymptomatic high-risk patients with limited life expectancy (CWC)	Endovascular repair of aneurysm, with diagnosis of abdominal aortic aneurysm without mention of rupture in the admission, and ASA code 4–5 or age 75 + with ASA 3. Exclude emergency admissions and admissions from the emergency department. Minimum age: 18. Sex: both	Endovascular repair of aneurysm, with diagnosis of abdominal aortic aneurysm without mention of rupture in the admission, and ASA code 4–5 or age 75 + . Minimum age: 18. Sex: both
Avoid performing an endoscopy for dyspepsia without alarm symptoms for patients under the age of 55 years (CWC)	Endoscopy with diagnosis of dyspepsia, and no diagnoses of dysphagia, iron deficiency anaemia, other nutritional anaemia, abnormal weight loss, personal or family history of cancer of digestive system, or personal history of peptic ulcer disease in the previous 12 months. Minimum age: 18. Maximum age: 54. Sex: both	Endoscopy with diagnosis of dyspepsia and no diagnoses dysphagia, iron deficiency anaemia, other nutritional anaemia, abnormal weight loss, personal or family history of cancer of digestive system, or personal history of peptic ulcer disease in the admission. Minimum age: 18. Maximum age: 54. Sex: both
Don’t perform fusion surgery to treat patients with mechanical axial low back pain from multilevel spine degeneration in the absence of: … (CWC)	Spinal fusion with diagnosis of low back pain with no mention of sciatica, spondylolisthesis, spinal deformities, or pain in legs in previous 12 months. Minimum age: 18. Sex: both	Spinal fusion with diagnosis of low back pain or spinal stenosis with no mention of sciatica, spondylolisthesis, spinal deformities, or pain in legs in admission. Minimum age: 18. Sex: both
Avoid recommending knee arthroscopy as initial/management for patients with degenerative meniscal tears and no mechanical symptoms (CWUS)	Knee arthroscopy in patients with knee osteoarthritis and no diagnosis of ligament strain or damage and no diagnosis of pyogenic arthritis. Minimum age: 55. Sex: both [27, 28]	Knee arthroscopy in patients with knee osteoarthritis or meniscal derangements and no diagnosis of ligament strain or damage and no diagnosis of pyogenic arthritis. Minimum age: 18. Sex: both [27, 28]
Don’t use epidural steroid injections (ESI) for patients with axial low back pain who do not have leg dominant symptoms originating in the nerve roots (CWC, CWA)	ESI with diagnosis of low back pain with no mention of leg pain or radiculopathy in previous 12 months. Minimum age: 18. Sex: both	ESI with diagnosis of low back pain with no mention of leg pain or radiculopathy in admission. Minimum age: 18. Sex: both
Avoid an open approach for primary bariatric surgical procedures [as opposed to a laparoscopic approach] (CWUS)	Bariatric procedure (including sleeve gastrectomy, gastric bypass, gastroplasty, gastric banding, biliopancreatic diversion, gastric reduction) and no previous bariatric procedure recorded within 12 months. No codes indicating revision or reversal procedure. No laparoscopic procedure codes in admission. Minimum age: 18. Sex: both	
Don’t routinely remove the gallbladder [during bariatric surgery] unless clinically indicated (CWUS)	Bariatric procedure with cholecystectomy in admission. No gallbladder disease in previous 12 months. Minimum age: 18. Sex: both	
Don’t use IVC filters as primary prevention of pulmonary emboli in the absence of an extremity clot or prior pulmonary embolus (CWUS) Don’t use inferior vena cava (IVC) filters routinely in patients with acute VTE (CWUS)	IVC insertion with no current or past pulmonary embolism (PE) diagnosis or deep vein thrombosis (DVT) in previous 12 months, or current acute venous thromboembolism. Minimum age: 18. Sex: both	All IVC insertions [16]
In general there is no indication to perform prophylactic retinal laser or cryotherapy to asymptomatic conditions such as lattice degeneration (with or without atrophic holes), for which there is no proven benefit (CWA) Do not carry out laser retinopexy for asymptomatic lattice degeneration/atrophic retinal holes (CWUK)	Retinal laser or cryotherapy procedure and lattice degeneration diagnosis, with no procedure code indicating repair of retinal detachment, or history of diagnosis of retinal detachment in previous 12 months. Minimum age: 18. Sex: both	Retinal laser or cryotherapy procedure and lattice degeneration diagnosis, with no procedure code indicating repair of retinal detachment, or history of diagnosis of retinal detachment. Minimum age: 18. Sex: both
If a child is under 12 months old and has a blocked nasolacrimal duct, do not try to unblock. (CWUK)	Lacrimal duct probing procedure on patient under 12 months, with diagnosis of stenosis of lacrimal duct in claim. Sex: both	Lacrimal duct probing procedure on patient under 12 months. Sex: both
Don’t perform endometrial biopsy in the routine evaluation of infertility (CWUS)	Endometrial biopsy, not related to suspicion of malignancy, with infertility diagnosis given as primary diagnosis. Minimum age: 18. Sex: female	Endometrial biopsy, not related to suspicion of malignancy, with infertility diagnosis. Minimum age: 18. Sex: female
Intravitreal injections may be safely performed on an outpatient basis. Don’t perform routine intravitreal injections in a hospital or day surgery setting unless there is a valid clinical indication (CWA)	Intravenous injection, not associated with other intraocular surgery or for a patient requiring anaesthetic services. Minimum age: 18. Sex: both	
Day surgery should be considered the default for most surgical procedures (except complex procedures). Variation in the use of day surgery for specific operations should be measured and this information should be available to patients (CW UK)		

*ASA* American Society of Anesthesiologists Physical Status Classification [29], *CWA* Choosing Wisely Australia, *CWC* Choosing Wisely Canada, *CWUK* Choosing Wisely United Kingdom, *CWUS* Choosing Wisely United States, *IVC* inferior vena cava, *VTE* venous thromboembolism

When developing the narrow indicator we used the most restrictive of any duplicate or similar recommendations, excluded procedures or diagnoses where appropriateness was unclear, and defined proxy measures to err towards counting care as appropriate. To develop the broader definition we included any ambiguous diagnosis or procedure codes that may capture inappropriate care, or adjusted the age, sex, or indications for which the service is potentially low-value.

#### Clinical review

Draft indicators were presented at a workshop involving 27 clinicians. Two authors (TBP and AGE) presented the overall approach, and invited feedback on the project as a whole. The clinicians then separated into groups where each reviewed 3 to 4 draft indicators. The clinicians were asked to take the CW recommendations as given (thus respecting the various CW processes for developing recommendations), and assess whether the narrow and broad indicators adequately captured the care targeted by the recommendation. They examined both the description of the indicator (as presented in Table 1) and the codes used in identifying low-value claims. We revised the indicators to incorporate the feedback, and a clinical coder reviewed the revised versions to assess the codes. Clinical co-authors (JB, IAS) resolved further questions about the indicators, or we occasionally consulted other specialists, including those present at the workshop.

### Results

**Fig. 2:**
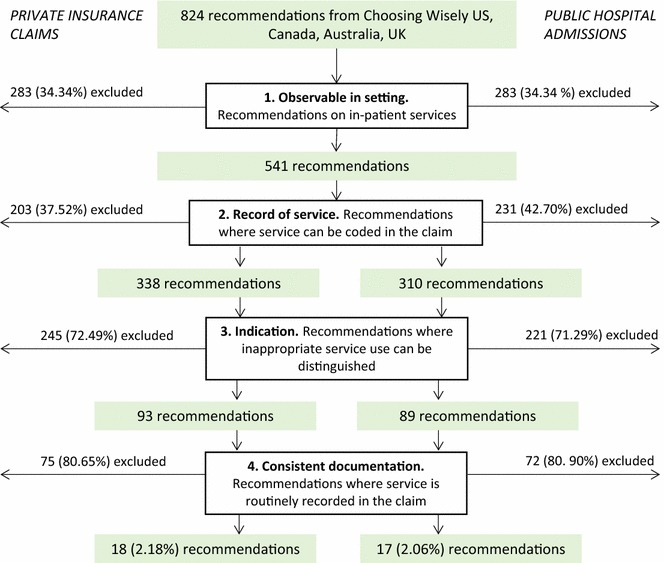
Recommendations excluded at each step, for public and private datasets. Recommendations were downloaded from the websites of the Choosing Wisely initiatives in the United States, Canada, Australia, and the United Kingdom in January 2017 [12–15]. All recommendations were then assessed against the four criteria in the Australian hospital inpatient setting and the private and public inpatient claims data settings. Differences in the measurable recommendations between private and public are due to differences in the variables held in the two datasets

We excluded 283 recommendations (34.3% of total) that were not *observable in setting* as they did not relate to inpatient services (Fig. 2). Then we excluded 203 (37.5%) of the remaining recommendations from measurement in the PHI data and 231 (42.7%) in the public hospital data, because there was no *record of service* in the datasets. These were mainly on pathology or prescribing services. The number excluded in the public and private hospital data differs because some services relate to an MBS item but not an ACHI code. For example, MBS items record inpatient intravitreal injections in PHI claims but not claims from public hospitals (and there is no specific ACHI code for this service).

Of the remaining recommendations, 93 (27.5%) met the *indication* criteria in the PHI data and 89 (28.7%) in the public hospital data, meaning we could identify a low-value indication for the service using the clinical details in the datasets.

Finally, we excluded 75 (81%) recommendations from the PHI data because the service was not *consistently documented* in the claims. We also excluded 72 (82%) from measurement in the public hospital data. This left 18 and 17 measurable recommendations in the PHI and public hospital datasets respectively (2.06 and 1.94% of all recommendations). Throughout this selection process, we resolved discrepancies for 68 recommendations (8.25% of all). The disagreement usually related to whether the recommendation was relevant in an inpatient setting or if the low-value indication was measurable.

We adapted 17 of the 18 measurable recommendations into indicators. Three pairs of similar or identical recommendations were combined into single indicators (Table 1). We omitted a UK recommendation that most surgical procedures should be a day surgery and “variation in the use of day surgery for specific operations should be measured”. Although it is measurable, adapting this specific recommendation will require considerable input from practitioners for exclusion criteria, depend on regional health systems’ day surgery policies, and could include many procedures. We therefore developed 14 indicators of low-value care.

During the clinical review process, participants suggested diagnoses justifying the procedure, thus making the indicators more specifically targeted at low-value care. They also advised against a strict age limit for “limited life expectancy”, resulting in a proxy measure based on age and American Society of Anesthesiologists risk score.

## Limitations

Eighteen of the 824 original recommendations were measurable in the datasets after excluding 807 (97.9%) recommendations. Similar studies have also found that most recommendations are not measurable in routinely collected data. Duckett et al. [18] identified 5 out of 1208 (0.4%) low-value services they could measure in hospital data. In primary care data, Sprenger et al. [19] measured 34 (2%) of 1658 recommendations. The small number of measurable recommendations is a consequence of the limited clinical information and health care setting coverage of these datasets (e.g. non-linked inpatient, primary care, pathology, radiology and pharmaceutical data). Using linked data may mean more recommendations are measurable in routinely collected data.

Our operational definitions of low-value care would benefit from further validation, such as comparison to a clinical chart review. This is a common issue when measuring low-value care in routinely collected data [20]. Validation studies using clinical chart review have, however, suggested either reasonable agreement or a conservative estimate of low-value care using the routine data approach [21–23].

These indicators are useful for examining variation and trends and to prioritise low-value care initiatives. CW is clinician-led and aims to facilitate discussions on appropriate care between doctors and patients, and not to proscribe services [24, 25]. Large questions still exist around the utility of auditing and feeding back low-value care measurement data to hospital managers and clinicians, recognising there is potential risk to the goodwill underlying Choosing Wisely type initiatives [24]. These indicators are useful, however, in facilitating questions and discussions regarding the true extent of low-value care, particularly where rates appear high within particular settings.

Our criteria allow systematic and transparent selection of recommendations for direct measurement within our data setting. The criteria and approach can be replicated in other data settings, including outside Australia, as well as other inappropriate care recommendations.

## Abbreviations

ACHI: Australian Classification of Health Intervention
ASA: American Society of Anesthesiologists
CT: computed tomography
CW: Choosing Wisely
CWA: Choosing Wisely Australia
CWC: Choosing Wisely Canada
CWUS: Choosing Wisely United States
DVT: deep vein thrombosis
MBS: Medical Benefits Scheme
NSW: New South Wales
PE: pulmonary embolism
PHI: private health insurance
UK: United Kingdom
US: United States
VTE: venous thromboembolism
